# Case Report: Switching secukinumab to bimekizumab in diffuse cutaneous systemic sclerosis after autologous hematopoietic stem cell transplantation: a case follow-up

**DOI:** 10.3389/fimmu.2026.1771650

**Published:** 2026-03-10

**Authors:** Lea-Kristin Nagler, Patrick-Pascal Strunz, Hannah Labinsky, Anja Kroiß, Michael Gernert, Marc Schmalzing

**Affiliations:** Department of Internal Medicine 2, Rheumatology/Clinical Immunology, University Hospital Würzburg, Würzburg, Germany

**Keywords:** bimekizumab, diffuse cutaneous systemic sclerosis, IL17A/IL-17F, interleukin-17, secukinumab, systemic sclerosis

## Abstract

Diffuse cutaneous systemic sclerosis (dcSSc) is a heterogeneous autoimmune disease characterized by progressive skin fibrosis, vasculopathy and variable organ involvement. Our recent case report about this patient described clinical improvement under IL-17A inhibition with secukinumab for worsening of cutaneous involvement after autologous hematopoietic stem-cell transplantation. We present the extended disease course through 2025. In July 2025 the patient presented with a new flare characterized by cutaneous tightening, dysesthetic sensory symptoms and intermittent pruritus despite ongoing secukinumab therapy. Given the early signs of renewed cutaneous progression and with the aim of preventing further deterioration, treatment was switched to bimekizumab, a dual IL-17A/IL-17F inhibitor. By November 2025 the patient demonstrated resolution of cutaneous symptoms, improvement of modified Rodnan skin score and improved joint stiffness. A single adverse event was mild oral candidiasis. This case follow-up suggests that broader IL-17 pathway inhibition may represent a promising therapeutic approach for selected patients with refractory inflammatory cutaneous manifestations of dcSSc.

## 1 Introduction

Diffuse cutaneous systemic sclerosis (dcSSc) is a multisystem autoimmune disease characterized by fibrosis, microvascular dysfunction and immune dysregulation affecting multiple organ systems (1). Skin thickening represents a major determinant of disease burden, and treatment options remain limited for patients with progressive or refractory cutaneous involvement. Off-label biologic therapies targeting cytokine pathways implicated in systemic inflammation - such as interleukin-17A (IL-17A) - have been explored in individual cases, and efficacy was further supported by a recent randomized phase 3 trial of the IL-17RA inhibitor brodalumab (2, 3). The original case report of this patient described an initial clinical improvement under secukinumab, an IL-17A monoclonal antibody, for cutaneous worsening early after autologous hematopoietic stem-cell transplantation (aHSCT) (4). However, long-term disease control remains challenging in dcSSc, and the potential role of broader IL-17 pathway inhibition - including IL-17F - has gained attention based on mechanistic, translational and clinical evidence from other inflammatory diseases. Bimekizumab, a monoclonal antibody targeting both IL-17A and IL-17F, has shown superior efficacy to secukinumab in several IL-17-mediated conditions (5–7). Here we provide an extended follow-up of the same patient after publication of the original report, focusing on the cutaneous flare during secukinumab treatment in mid-2025 and subsequent clinical response after switching to bimekizumab.

## 2 Case presentation

**Figure 1:**
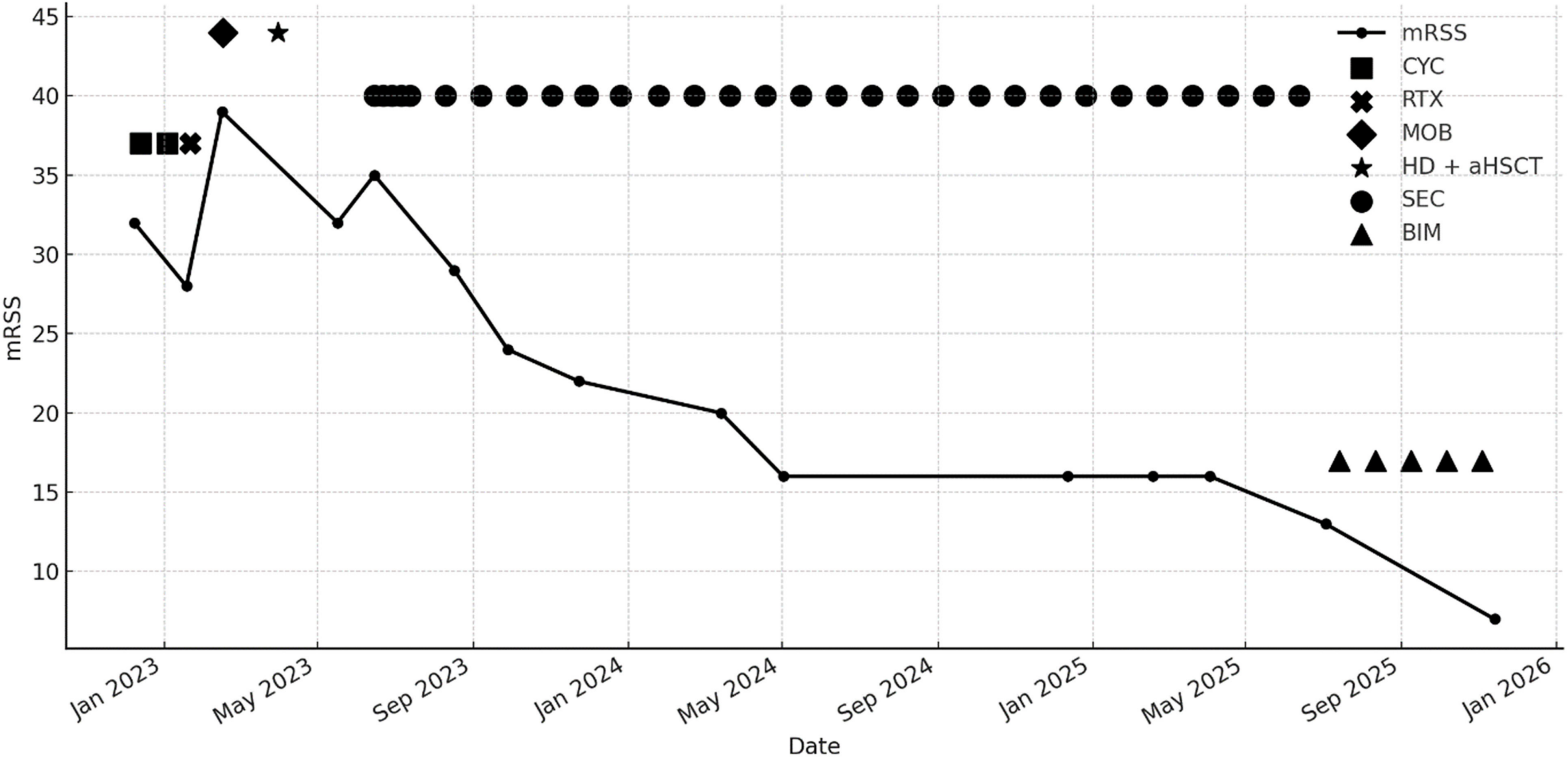
Modified Rodnan Skin Score (mRSS) over time in relation to the treatment regimen from December 2022 to November 2025. All mRSS assessments were performed by the same experienced investigator. The y-axis displays the mRSS values, plotted longitudinally across the observation period, treatment events are indicated by distinct symbols. The Figure was generated analogously to our first published case report (3). aHSCT autologous haematopoietic stem-cell transplantation; BIM, bimekizumab; CYC, cyclophosphamide; HD, high-dose chemotherapy; MOB, mobilization chemotherapy; RTX, rituximab; SEC, secukinumab.

The patient is a 43-year-old woman with Scl-70–positive, rapidly progressive diffuse systemic sclerosis (dcSSc), initially diagnosed in 2021 with pulmonary and cardiac involvement. In December 2022 she was admitted for initiation of immunosuppressive therapy, presenting with a modified Rodnan Skin Score (mRSS) of 32 and preserved pulmonary function (FVC 79% predicted). Despite cyclophosphamide therapy, she experienced a severe flare in January 2023 with rapid skin progression, stiffness and functional impairment, prompting indication for autologous hematopoietic stem-cell transplantation. Also, a bridging therapy with rituximab was administered due to the rapid disease progression. After mobilization chemotherapy and stem-cell harvest, she developed progressive renal failure interpreted as scleroderma renal crisis, requiring antihypertensive therapy and later hemodialysis. Given continued aggressive skin progression (mRSS 39, **Figure 1**) and contraindications to other immunosuppressive strategies, aHSCT was performed using melphalan and ATG conditioning. Post-transplantation, initial improvement was observed (mRSS 32), yet a new flare in June 2023 revealed recurrent diffuse skin sclerosis (mRSS 35, **Figure 1**). As prior rituximab treatment had been ineffective and peripheral B cells remained fully depleted, further B-cell–targeted therapies were not considered beneficial. Due to end-stage kidney failure requiring dialysis, additional immunosuppressants were contraindicated; thus secukinumab, an IL-17A inhibitor, was initiated in June 2023. Secukinumab was given subcutaneously with standard loading dose of 300 mg in week 0, 1, 2, 3, 4 followed by four-weekly administration. Peripheral B-cell reconstitution was defined by reappearance of peripheral CD19+ B cells and was first documented in August 2023 by flow cytometry. T-cell reconstitution was assessed by recovery of naïve CD4+ T cells and was observed in May 2024.

By August and September 2023, the patient showed marked clinical improvement with substantial skin softening (mRSS 24, **Figure 1**), improved range of motion and stable lung function. No adverse effects occurred under secukinumab. Importantly, the patient remained clinically stable throughout 2024 and into mid-2025, with sustained skin improvement (mRSS 13, **Figure 1**), stable organ involvement and progressive recovery of functional capacity (**Table 1**), allowing her to participate in daily life activities again. Notably, dialysis was no longer required from November 2023 onward, further contributing to her functional stabilization. Moreover, there was no evidence of ongoing microangiopathy. Haptoglobin levels had normalizes, fragmented erythrocytes were no longer detectable, and no further signs of hemolysis were present. Blood pressure was well controlled under antihypertensive medication. Therefore, the dose of secukinumab was reduced to 150 mg every four weeks in September 2023. This favorable course persisted until July 2025, when she developed a new symptomatic flare with progressively increasing skin tightness. Additional symptoms included mild general malaise and dysesthetic sensory disturbances, described as tingling, stinging and burning in the hands and feet, similar in pattern to early stages of previous flares. Short episodes of intermittent pruritus and slightly increased morning joint stiffness were reported. There were no new digital ulcers or respiratory symptoms, and thus far, no objective progression of skin fibrosis was detectable by mRSS at this early stage of symptom recurrence. Alternative dermatologic diagnoses including psoriasis or other inflammatory dermatoses, were excluded based on repeated clinical assessment.

**Table 1:** Disease parameters between November 2021 (primary diagnosis) and November 2025.

Disease parameters	11/21	12/22	01/23	02/23	03/23	05/23	06/23	08/23	09/23	12/23	04/24	08/24	12/24	04/25	07/25	11/25
Therapy		CYC	RTX	MOB	aHSCT		SEC	SEC	SEC	SEC	SEC	SEC	SEC	SEC	BIM	BIM
FVC predicted	89%	n. d.	79%	n. d.	n. d.	n. d.	n. d.	79%	n. d.	n. d.	81%	95%	94%	94%	86%	n. d.
DLCO_SB	61%	n. d.	37%	n. d.	n. d.	n. d.	n. d.	36%	n. d.	n. d.	54%	51%	58%	57%	56%	n. d.
eGFR (MDRD) [ml/min]	80	99	94	104	30	6	dialysis	dialysis	dialysis	32	35	39	34	34	31	37
hs Troponin [pg/ml]	n. d.	58.3	138.4	n. d.	n. d.	393.0	121.0	61.8	40	25	24.3	12.1	27.0	8.9	14.1	9.3
sPAP [mmHg]	n. d.	n. d.	21.51	n. d.	n. d.	26.0	n. d.	n. d.	n. d.	21.98	n. d.	30.26	n. d.	20.43	n. d.	n. d.
Haptoglobin [md/dl]	n. d.	154	171	166	<10	205	138	122	87	152	114	119	104	93	169	92
Fragmented erythrocytes	n. d.	n. d.	n. d.	n. d.	0,7%	n. d.	n. d.	n. d.	n. d.	0%	n. d.	n. d.	n. d.	n. d.	0%	n. d.
Bilirubin [mg/dl]	n. d.	0.3	0.2	0.3	0.6	0.2	0.2	0.2	0.4	0.3	0.6	0.3	0.3	0.4	0.5	0.5

aHSCT autologous hematopoietic stem-cell transplantation; BIM, bimekizumab; CYC, cyclophosphamide; DLCO_SB, single-breath carbon monoxide diffusing capacity of the lung; eGFR, estimated glomerular filtration rate; FVC, forced vital capacity; HD, high-dose chemotherapy; hs, high sensitive; MOB, mobilization chemotherapy; n.d, not done; RTX, rituximab; SEC, secukinumab; sPAP, systolic pulmonary artery pressure.

A trial of levocetirizine was attempted for pruritus but remained ineffective over two weeks. Because the patient had previously shown a sustained and robust clinical response to secukinumab monotherapy, and the newly emerging cutaneous progression was recognized at an early stage, the treatment strategy was revised and therapy was switched to bimekizumab, also administered as monotherapy at a dose of 320 mg subcutaneously at weeks 0, 4, 8, 12, 16, followed by 320 mg every eight weeks without further immunosuppressive treatment.

Under bimekizumab, the patient experienced rapid and substantial improvement. By November 2025, she reported near-complete resolution of skin tightness, disappearance of neuropathic sensory symptoms and reduced joint stiffness. Physical examination confirmed softening of previously affected skin and stable cardiopulmonary findings (mRSS 7, **Figure 1**, **Table 1**). The only treatment-related event was a mild episode of oral candidiasis, which resolved by fluconazole treatment.

## 3 Discussion

This extended follow-up provides further insight into the potential role of IL-17 pathway modulation in selected cases of inflammatory, skin-dominant systemic sclerosis. While IL-17A has been implicated in experimental models of endothelial and fibroblast activation (8, 9), and elevated levels of several IL-17 family cytokines have been reported in SSc cohorts (10, 11), the precise contribution of IL-17A versus IL-17F remains incompletely understood. Secukinumab, which selectively targets IL-17A, achieved a sustained and meaningful clinical response in this patient for an extended period. The subsequent flare occurring despite continued IL-17A inhibition, however, suggests that additional cytokines - particularly IL-17F - may drive inflammatory activity during certain disease phases and could necessitate broader pathway blockade, a concept supported by mechanistic and clinical data demonstrating superior skin efficacy of dual IL-17A/IL-17F inhibition (5, 6).

Beyond this single case, early clinical trials have explored IL-17 pathway inhibition in SSc. A phase 1 single-arm open-label trial of the IL-17RA antagonist brodalumab in early diffuse cutaneous SSc reported a rapid and significant reduction in mRSS, along with improvements in dermal thickness and digital ulcer mean counts, although pulmonary function remained unchanged (2). These findings suggest that IL-17 pathway modulation may impact cutaneous and microvascular manifestations in SSc, though its effect on major internal organ involvement (such as lung, heart or kidneys) remains unclear.

Bimekizumab neutralizes IL-17A- and IL-17F-monodimers, as well as IL-17A/F heterodimers, thereby achieving broader suppression of the IL-17 pathway. In psoriasis, a head-to-head trial demonstrated significantly superior skin clearance with bimekizumab compared with secukinumab, supporting the notion that IL-17F acts as an important amplifier of IL-17–driven cutaneous inflammation (6). Although psoriasis and systemic sclerosis differ pathogenetically, inflammatory skin manifestations in SSc may share IL-17–mediated components that render broader cytokine inhibition advantageous.

The patient’s rapid and near-complete symptom resolution after switching to bimekizumab mirrors the deeper responses observed in IL-17–mediated diseases treated with dual IL-17A/IL-17F blockade (5–7). The mild episode of oral candidiasis aligns with known class effects of IL-17 pathway inhibition, as IL-17F blockade modestly increases susceptibility but events are generally manageable (7).

Although aHSCT can induce sustained improvement in diffuse systemic sclerosis, disease relapse occurs in a subset of patients, particularly those with severe or rapidly progressive disease (12, 13). Interindividual variability in immune reconstitution after aHSCT may allow re-emergence of pathogenic immune pathways. In addition, cytokine-driven inflammation, including IL-17–related pathways, may persist despite immune reset (14, 15).

This report has several limitations. The flare prompting therapeutic adjustment was defined clinically - based on early symptoms such as tightening, dysesthesia and pruritus - without an accompanying increase in mRSS, which reduces the objectivity of flare assessment. In addition, no skin biopsies were obtained, preventing histopathological correlation. The findings derive from a single case with limited follow-up. Moreover, potential delayed effects of the preceding aHSCT cannot be fully excluded and may have contributed to the observed clinical improvement.

While causality cannot be established from a single case, this follow-up suggests that dual IL-17A/IL-17F blockade may offer more effective control of cutaneous disease activity in diffuse systemic sclerosis than IL-17A inhibition alone. Systematic clinical studies are therefore needed to further clarify the role of IL-17 family cytokines in SSc pathogenesis and to evaluate the therapeutic potential of broader IL-17 pathway inhibition.

## 4 Conclusion

In this case of diffuse systemic sclerosis, secukinumab provided a clinically relevant improvement of skin manifestation for an extended period before cutaneous symptoms reappeared. Escalation to bimekizumab resulted in a rapid and marked clinical improvement with acceptable tolerability. This follow-up suggests that broader IL-17 pathway inhibition may provide superior control of inflammatory cutaneous manifestations in selected cases of dcSSc and underscores the rationale for further studies of dual IL-17A/IL-17F blockade in SSc.

## Data Availability

The raw data supporting the conclusions of this article will be made available by the authors, without undue reservation.
